# Temporal costs of access to formal health care among adult users in Peru: a national analysis of inequalities in travel and waiting times, 2016–2024

**DOI:** 10.3389/frhs.2026.1864862

**Published:** 2026-06-22

**Authors:** Víctor Juan Vera-Ponce, Jhosmer Ballena-Caicedo, Lily Mabel Portal-Valqui, Adriana Mishell Yoplac-Oyarce, Kevin Cusma-Regalado, Namibia Jherly Cervera Vasquez, Fiorella E. Zuzunaga-Montoya

**Affiliations:** Facultad de Medicina (FAMED), Universidad Nacional Toribio Rodríguez de Mendoza de Amazonas (UNTRM), Chachapoyas, Peru

**Keywords:** access to health services, geographic accessibility, health inequities, national surveys, Peru, waiting time

## Abstract

**Introduction:**

The time spent accessing health care, both in travel and in waiting, represents a poorly monitored dimension of inequity in health systems. This study estimated territorial and socioeconomic inequalities in travel and waiting times in Peru from 2016 to 2024 among adults who attended formal health care facilities.

**Methods:**

We analyzed nine rounds of the Peruvian National Household Survey (ENAHO) using a repeated cross-sectional design. The main sample included adults who attended formal facilities and had valid simultaneous information on travel time and waiting time. The outcomes were travel time >60 min, waiting time >60 min, and poor access, defined as both travel and waiting times >60 min. Weighted prevalences and adjusted prevalence ratios (aPRs) were estimated.

**Results:**

The prevalence of travel time >60 min was 4.0% in Metropolitan Lima, 4.2% in other urban areas, and 11.3% in rural areas (rural vs. Lima aPR: 3.32; 95% CI: 2.92–3.79). The pattern was reversed for waiting time >60 min: 22.2% in Metropolitan Lima, 21.8% in other urban areas, and 9.2% in rural areas (rural aPR: 0.40; 95% CI: 0.37–0.43). Poor access was uncommon but showed a consistent territorial gradient. Acceptable waiting time and optimal access improved through 2022 and then declined in 2023–2024.

**Discussion:**

Among adult users who successfully accessed formal facilities, temporal inequities in Peru operate in opposite directions: rural disadvantage is concentrated in travel time, whereas urban disadvantage is concentrated in waiting time. Equity policies should distinguish geographic from organizational barriers and incorporate time-based indicators into routine health-system monitoring.

## 1 Introduction

Universal health coverage remains an unfinished goal worldwide. The joint monitoring report by the World Health Organization (WHO) and the World Bank showed that progress in service coverage has stagnated since 2015 and that catastrophic spending continues to affect large segments of the population (1). However, discussions of access cannot be limited to the existence of financial coverage or to the mere use of services. The literature on high-quality health systems has emphasized that effective access requires timeliness, continuity, problem-solving capacity, and care experience, beyond simple contact with the system (2). Within this framework, time becomes a particularly important dimension: prolonged waiting functions as a form of cost transferred to the patient and as an indicator of health systems under strain (3).

In Latin America, efforts to expand coverage have coexisted with fragmented, segmented, and unequal systems. In Peru, this situation is reflected in the coexistence of subsystems with different financing arrangements, provider networks, and benefit packages, including the Ministry of Health, the Social Health Insurance system (EsSalud), the Armed Forces and Police services, and the private sector (4). Regional studies have shown that access barriers and inequities in quality persist even in settings with high nominal coverage (5, 6). In Peru, institutional fragmentation, underinvestment, and problems in budget management have been identified as structural determinants of poor health-system performance and persistent access gaps (4, 7).

The geographic dimension of access is especially relevant in Peru because of its territorial diversity and the heterogeneity of its health infrastructure. A national geospatial study estimated that travel times to primary, secondary, and tertiary facilities were substantially longer in rural than in urban areas (8). Similarly, among Indigenous communities in the Peruvian Amazon, a substantial proportion were located more than one hour from the nearest facility (9). At the same time, the organizational dimension of access is also important: in Peruvian ambulatory care facilities, longer waiting times are associated with lower user satisfaction (10). These two dimensions—travel and waiting—represent distinct stages of the patient pathway and may reveal different inequities.

Recent Peruvian evidence has focused mainly on non-use of services, barriers to seeking care, socioeconomic inequality in medical consultations, and the effect of the pandemic on unmet health care needs (5, 11, 12). Less is known, however, about how the temporal cost of care is distributed among those who do manage to use formal services, and whether inequalities are similar or divergent according to the stage of access being evaluated. Therefore, this study aimed to examine territorial and socioeconomic inequalities in travel and waiting times in Peru from 2016 to 2024 among adults who attended formal health care facilities, and to describe the temporal evolution of these indicators.

## 2 Materials and methods

### 2.1 Study design and data source

We conducted an analytical observational study using repeated cross-sectional secondary data from the Peruvian National Household Survey (ENAHO) for 2016–2024, using the public databases and documentation of the National Institute of Statistics and Informatics (INEI) (13). ENAHO is an annual population-based multipurpose survey conducted by Peru's National Institute of Statistics and Informatics. Each annual round uses a probabilistic, stratified, multistage sampling design with national and area-of-residence representativeness. For this study, nine independent annual rounds were pooled for two purposes: to increase the precision of the estimates and to describe the temporal evolution of access indicators among users of formal health services. The study was reported following the Strengthening the Reporting of Observational Studies in Epidemiology (STROBE) guideline for observational studies (14). The corresponding checklist is presented as Supplementary Table S1.

The ENAHO health module collects information on recent morbidity, care seeking, place of care, travel time, waiting time, and services received during the four weeks before the interview. The unit of analysis was the interviewed adult. This study focused on the access experience of people who actually attended formal health care facilities, rather than on health care need in the general population.

### 2.2 Study population, selection criteria, and definition of analytical samples

The source population comprised individual adult records available in ENAHO 2016–2024. From this universe, we identified individuals who reported receiving care at a formal health care facility during the four weeks before the interview. Formal facilities were defined as Ministry of Health health posts and health centers, CLAS facilities, Ministry of Health hospitals, EsSalud health posts and hospitals, Armed Forces or National Police hospitals, private medical offices, and private clinics. Care reported in pharmacies or drugstores, at home, or in other noninstitutional settings was excluded because it does not represent the same stage of the access process as care delivered in formal facilities.

Accordingly, the target estimand was not health-care access among all adults with health care needs, but the temporal burden experienced by users who crossed the threshold into formal facility-based care.

Two analytical samples were defined. The main analytical sample was used for time-based outcomes and required valid simultaneous information on travel time and waiting time. The supplementary sample was reserved for the analysis of non-receipt of medications and was defined among participants who reported receiving a medical consultation and had non-missing information on receipt of medications. In both cases, missing values were not imputed; therefore, analyses were based on complete cases within each analytical sample. No formal sample-size calculation was performed because all eligible adult records available in the nine analyzed rounds were included; precision was assessed using confidence intervals that incorporated the complex sampling design.

The flow diagram of the selection process is shown in Supplementary Figure S1. In brief, of 735,626 individual records available in ENAHO 2016–2024, 125,429 corresponded to adults who attended a formal health care facility. Of these, 95,002 formed the main analytical sample with valid simultaneous information on travel and waiting times; therefore, 30,427 records (24.3% of eligible formal-service users) were not included in the main sample because of incomplete temporal information or information recoded as missing. In addition, 10,583 participants formed the supplementary medication sample.

### 2.3 Variable construction

#### 2.3.1 Main outcomes

The main outcomes were based on two temporal dimensions of access: travel time and waiting time. Travel time was calculated in minutes from the reported hours and minutes for the trip from home to the health care facility. Waiting time was calculated in minutes from the reported hours and minutes between arrival at the facility and receipt of care.

Unacceptable travel time was defined as a time greater than 60 min, and excessive waiting time as a time greater than 60 min. Complementary mirror variables—travel ≤60 min and waiting ≤60 min—were generated to facilitate the presentation of temporal trends. A composite indicator of optimal access was also constructed when both dimensions were ≤60 min, and a composite indicator of poor access when both dimensions were >60 min. The 60-minute cutoff was defined *a priori* because it is programmatically interpretable and consistent with the one-hour threshold used in previous Peruvian studies of geographical access to health facilities (8, 9). For waiting time, the same threshold was applied to maintain a symmetric operational definition of excessive temporal burden across the two stages of access. Thus, this cutoff should be interpreted as a transparent operational threshold, not as a universal clinical standard.

Values greater than 480 min (eight hours) in either temporal dimension were treated as extreme values and recoded as missing for the main analysis, following an *a priori* rule to reduce the influence of potentially erroneous reports. This decision prevented observations with extreme times from distorting the estimates, without unnecessarily excluding individuals from other analyses to which they could still contribute valid information.

#### 2.3.2 Supplementary outcome

As a supplementary outcome, we assessed non-receipt of medications among participants who reported receiving a medical consultation. This variable was defined as not having received medications among people with a recorded medical consultation and available information for this component. Because this outcome has a different clinical denominator from the time-based outcomes, it was not incorporated into the main composite index and was analyzed separately.

#### 2.3.3 Explanatory variables and covariates

The main independent variable was detailed area of residence, classified as Metropolitan Lima, other urban, and rural according to the official ENAHO stratification. Socioeconomic status was approximated using household per capita expenditure quintiles, defined within each survey year to preserve households’ relative position in each annual round.

The organization of care was summarized using the health subsector, classified as Ministry of Health, EsSalud, private, and Armed Forces/Police. To construct this variable, the reported type of facility was first identified. When the same participant selected more than one formal category, a fixed hierarchy was applied to assign a single predominant facility, and this classification was then collapsed into the four analytical subsectors. This decision produced a mutually exclusive and reproducible variable for multivariable models.

Adjustment covariates included sex, age, and educational attainment. Age was included as a continuous variable in adjusted models and, for descriptive purposes, was summarized in the observed categories of 18–59 years and 60 years or older. Educational attainment was classified as no formal education, primary, secondary, and higher education. Survey year was included as a categorical variable in the main models to capture nonlinear temporal variation.

Natural region and insurance status were not included in the final adjusted models. Natural region was excluded to maintain a parsimonious model centered on the territorial dimension most directly interpretable for service delivery, whereas insurance status was not added because it conceptually overlaps with the care subsector used for the analyzed episode and could induce overadjustment of the organizational differences that the study aimed to describe.

### 2.4 Statistical analysis

All analyses incorporated the complex sampling design of ENAHO by specifying clusters as primary sampling units, selection strata, and population expansion factors. For the pooled 2016–2024 analysis, annual expansion factors were divided by the number of survey rounds because the target estimand was the average experience of formal-service users across the pooled period, not the sum of nine annual populations. This constant rescaling preserves weighted proportions and model contrasts while avoiding artificial inflation of pooled population totals. For annual trends, each year was interpreted within its own survey round; stratum and cluster identifiers were made unique by year before specifying the pooled survey design.

The main analytical sample was described using unweighted absolute counts and weighted percentages according to sociodemographic characteristics and organization of care. For each binary outcome, weighted prevalences with 95% confidence intervals were estimated and disaggregated by area of residence. Annual trends in the main indicators were described using weighted prevalences for each year from 2016 to 2024. The absolute rural–urban gap in travel time >60 min was calculated as the difference between the rural and urban weighted prevalences for each year of the period.

Associations between covariates and each binary outcome were estimated using Poisson regression models with a log link and robust linearized variance, using the survey prefix to preserve the complex design structure. This approach was selected because it allows estimation of directly interpretable adjusted prevalence ratios and avoids the overestimation that logistic regression can produce when outcomes are frequent, as occurred particularly with waiting time >60 min.

The main models simultaneously included detailed area of residence, household per capita expenditure quintile, health subsector, continuous age, sex, educational attainment, and survey year. The three main outcomes—travel >60 min, waiting >60 min, and poor access—were modeled in the same main analytical sample to maximize comparability across tables, figures, and adjusted estimators. Multivariable modeling was performed on observations with complete information for all included covariates. The analysis of non-receipt of medications was conducted separately in its own subsample and is presented only as supplementary material.

To assess the potential implications of the complete-case requirement, we descriptively compared eligible formal-service users included in the main analytical sample with those excluded because of incomplete or invalid temporal information. This comparison is presented in Supplementary Table S2.

Adjusted prevalence ratios with 95% confidence intervals were estimated. Interpretation prioritized the magnitude and precision of the estimates rather than dichotomous statistical significance. All analyses were performed in Stata version 17, and tables and figures were prepared in R.

### 2.5 Ethics statement

The study was conducted in accordance with the principles of the Declaration of Helsinki and its subsequent amendments. ENAHO is a public, anonymized, open-access survey administered by the National Institute of Statistics and Informatics (INEI) (13), and its public microdata system provides databases and documentation while protecting statistical confidentiality. This study was a secondary analysis of de-identified public-use microdata; it did not involve recruitment, intervention, direct contact with participants, access to identifiable information, or linkage to external identifiable records. For this reason, and in accordance with local legislation and institutional requirements, ethical review and approval by an institutional research ethics committee were not required. This position is consistent with ethical guidance for secondary data analysis, which recognizes that the ethical obligation in such studies is centered on lawful data access, confidentiality protection, appropriate use of the existing dataset, and avoidance of participant re-identification rather than on obtaining a new approval for public anonymized data (15). Primary ENAHO data collection is conducted under INEI procedures for informed consent and confidentiality. Only aggregate estimates are reported, and no individual-level information or small-cell cross-tabulations for potentially identifiable subgroups were disclosed.

## 3 Results

### 3.1 Participant selection and analytical sample

Supplementary Figure S1 summarizes the selection process. In total, 735,626 individual records were identified in ENAHO 2016–2024. Of these, 125,429 corresponded to adults who reported receiving care at formal health care facilities. The main analytical sample comprised 95,002 participants with valid simultaneous information on travel time and waiting time; therefore, 30,427 records (24.3% of eligible formal-service users) were not included in this sample because of incomplete temporal information or information recoded as missing. The supplementary subsample for the medication analysis included 10,583 participants with a medical consultation and available information on receipt of medications.

Supplementary Table S2 compares eligible formal-service users included in and excluded from the main complete-case analytical sample. Compared with included records, excluded records showed higher weighted proportions in 2020–2021, Metropolitan Lima, higher expenditure and educational categories, and private-sector care. These differences support interpreting the main estimates as complete-case estimates conditional on valid simultaneous travel and waiting time information.

### 3.2 Characteristics of the main sample

Table 1 presents the characteristics of the main analytical sample. Of the 95,002 participants, 63.3% were women and 36.7% were men. In territorial terms, 48.3% resided in Metropolitan Lima, 26.2% in other urban areas, and 25.5% in rural areas. The socioeconomic distribution showed greater representation of the poorest quintile (25.1%), followed by Q2 (21.1%) and Q3 (19.9%), whereas the richest quintile accounted for 15.7% of the sample.

**Table 1:** Characteristics of the main analytical sample.

Characteristic	n	Weighted %
Main analytical sample	95,002	100.0
Sex		
Female	59,623	63.3
Male	35,379	36.7
Age		
18–59 years	62,275	65.6
≥60 years	32,727	34.4
Area of residence		
Metropolitan Lima	29,593	48.3
Other urban	25,956	26.2
Rural	39,453	25.5
Per capita expenditure quintile		
Q1 (poorest)	28,804	25.1
Q2	21,340	21.1
Q3	17,379	19.9
Q4	15,077	18.2
Q5 (richest)	12,402	15.7
Health subsector		
MINSA	67,590	67.2
EsSalud	24,223	28.8
Private	2,144	2.6
Armed Forces/Police	1,045	1.4
Educational attainment		
No formal education	9,218	8.5
Primary	32,712	31.0
Secondary	31,097	35.8
Higher education	21,968	24.6

n values are unweighted counts; percentages incorporate the complex sampling design and expansion factors. Educational attainment had 7 missing values; therefore, the counts in that section sum to 94,995.

MINSA, Ministry of Health; FF. AA., Armed Forces.

Regarding place of care, the Ministry of Health accounted for the largest proportion of analyzed episodes (67.2%), followed by EsSalud (28.8%), the private subsector (2.6%), and the Armed Forces/Police (1.4%). Regarding educational attainment, 35.8% reported secondary education, 31.0% primary education, 24.6% higher education, and 8.5% no formal education. Most participants were 18–59 years old (65.6%), whereas 34.4% were 60 years or older.

### 3.3 Prevalence of the main outcomes by area of residence

Supplementary Table S3 summarizes the weighted prevalence of the main outcomes by area of residence, and Supplementary Figure S2 provides a complementary graphical representation. Travel time >60 min showed marked territorial inequality: prevalence was 4.0% in Metropolitan Lima, 4.2% in other urban areas, and 11.3% in rural areas. In contrast, waiting time >60 min showed the opposite pattern, with prevalences of 22.2% in Metropolitan Lima, 21.8% in other urban areas, and 9.2% in rural areas.

The composite outcome of poor access was less frequent than its individual components, but maintained a consistent territorial gradient: 1.1% in Metropolitan Lima, 1.5% in other urban areas, and 1.9% in rural areas. Overall, these results show that inequities were not expressed in the same way across all access dimensions: the main rural disadvantage was concentrated in travel, whereas the main disadvantage in Lima and other urban areas was concentrated in waiting.

### 3.4 Temporal trends

**Figure 1:**
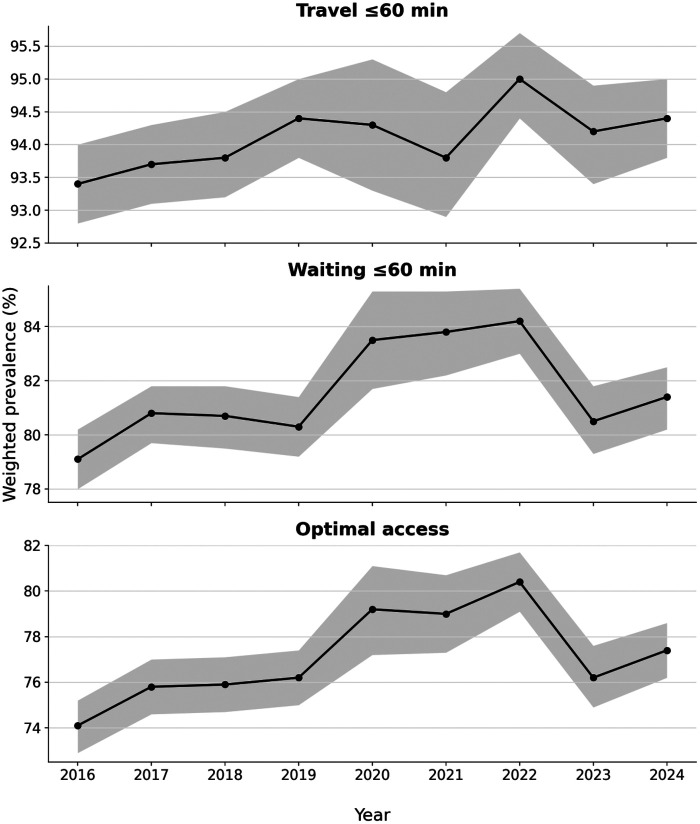
Annual trends in travel <=60 min, waiting <=60 min, and optimal access. Shaded bands represent 95% CIs.

Supplementary Table S4 and Figure 1 show the annual trends in the main indicators from 2016 to 2024. The percentage of participants with travel ≤60 min remained high and relatively stable throughout the period: 93.4% in 2016, 94.4% in 2019, 95.0% in 2022, and 94.4% in 2024. By contrast, waiting ≤60 min showed more visible improvement through 2022, increasing from 79.1% in 2016 to 84.2% in 2022, followed by a decline to 80.5% in 2023 and a partial recovery to 81.4% in 2024.

Optimal access, defined as travel and waiting ≤60 min, followed a trajectory similar to that of waiting time. The weighted prevalence increased from 74.1% in 2016 to 80.4% in 2022 and then decreased to 76.2% in 2023, with a slight recovery to 77.4% in 2024. Taken together, these series suggest that temporal changes in access were driven mainly by the waiting-time dimension and that the evolution over the period was not linear.

### 3.5 Urban–rural gap in travel >60 min

**Figure 2:**
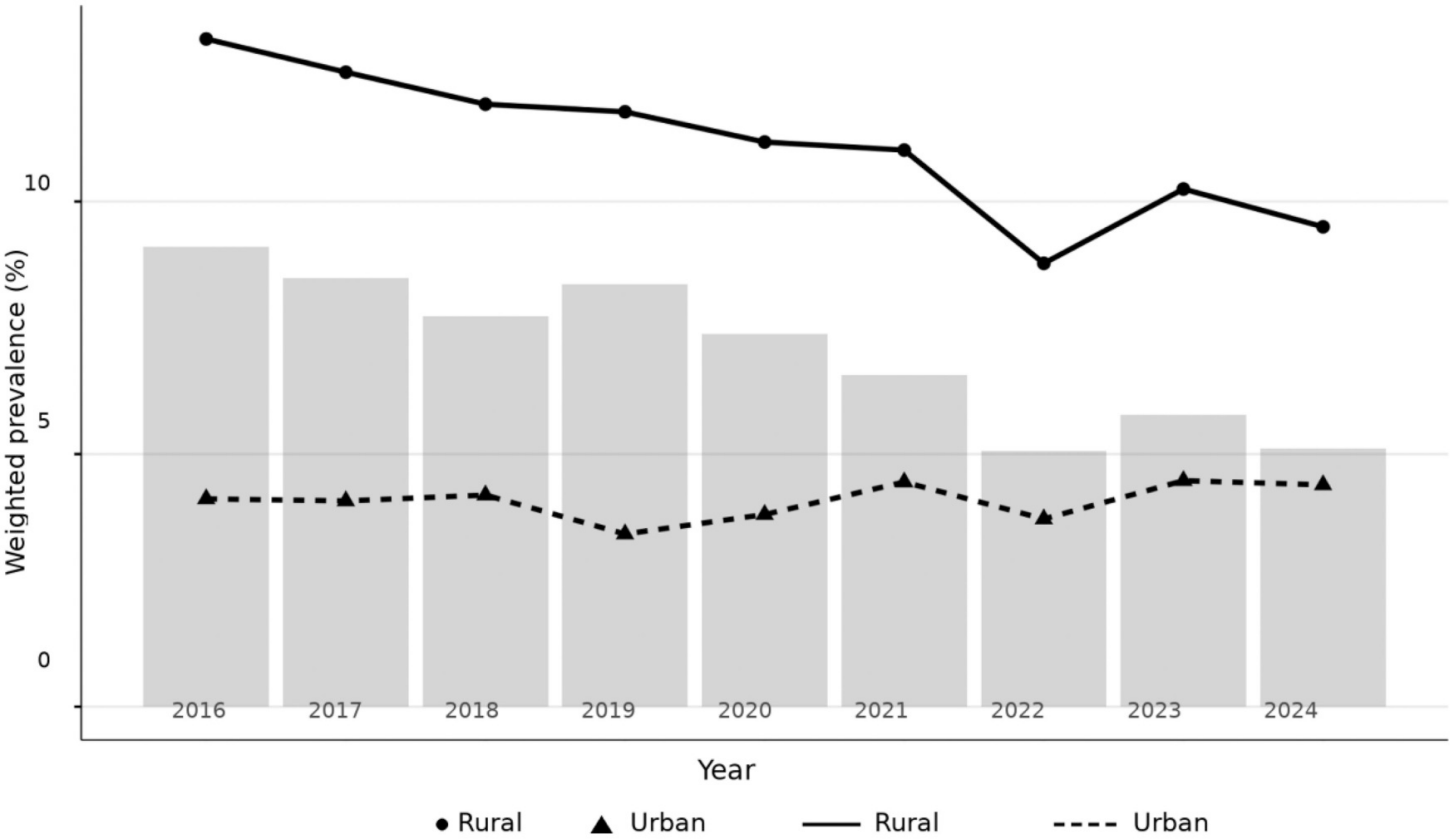
Urban-rural gap in travel >60 min. Bars represent the absolute rural-urban gap in percentage points, and lines show annual weighted prevalences.

Figure 2 shows the annual evolution of the rural–urban gap in travel time >60 min. Throughout the period, rural prevalence was consistently higher than urban prevalence. The absolute gap tended to narrow over time, from approximately 9 percentage points at the beginning of the period to approximately 5 percentage points in 2024. However, this reduction did not eliminate the inequity, because the rural excess in travel time persisted across all analyzed years.

### 3.6 Adjusted models of the main outcomes

Table 2 presents the adjusted prevalence ratios for the main outcomes, and Supplementary Figure S3 graphically summarizes the same results. For travel >60 min, other urban areas had a 16% higher prevalence than Metropolitan Lima (aPR 1.16; 95% CI 1.01–1.33), whereas rural areas had a prevalence more than three times higher (aPR 3.32; 95% CI 2.92–3.79). In the socioeconomic gradient, only the richest quintile had a lower prevalence of travel >60 min compared with the poorest quintile (aPR 0.77; 95% CI 0.64–0.92). By subsector, the prevalence of travel >60 min was higher in EsSalud (aPR 1.66; 95% CI 1.47–1.88), in the private subsector (aPR 1.60; 95% CI 1.29–1.99), and especially in the Armed Forces/Police (aPR 5.71; 95% CI 4.29–7.60), all compared with the Ministry of Health.

**Table 2:** Adjusted prevalence ratios for the main outcomes.

Characteristic	Travel >60 min aPR (95% CI)	Waiting >60 min aPR (95% CI)	Poor access aPR (95% CI)
Area of residence			
Metropolitan Lima (reference)	1.00 (ref.)	1.00 (ref.)	1.00 (ref.)
Other urban	1.16 (1.01–1.33)	0.96 (0.91–1.01)	1.46 (1.15–1.87)
Rural	3.32 (2.92–3.79)	0.40 (0.37–0.43)	2.07 (1.59–2.70)
Per capita expenditure quintile			
Q1 (poorest; reference)	1.00 (ref.)	1.00 (ref.)	1.00 (ref.)
Q2	0.93 (0.85–1.02)	1.13 (1.07–1.21)	1.12 (0.90–1.38)
Q3	0.93 (0.82–1.05)	1.13 (1.06–1.22)	1.23 (0.93–1.61)
Q4	0.93 (0.81–1.07)	1.10 (1.02–1.19)	1.04 (0.76–1.43)
Q5 (richest)	0.77 (0.64–0.92)	0.97 (0.89–1.05)	0.86 (0.59–1.24)
Health subsector			
MINSA (reference)	1.00 (ref.)	1.00 (ref.)	1.00 (ref.)
EsSalud	1.66 (1.47–1.88)	0.95 (0.90–1.00)	1.65 (1.26–2.16)
Private	1.60 (1.29–1.99)	1.00 (0.88–1.13)	1.48 (0.91–2.39)
Armed Forces/Police	5.71 (4.29–7.60)	0.68 (0.56–0.84)	4.18 (2.43–7.17)

aPR, adjusted prevalence ratio; 95% CI, 95% confidence interval; MINS, ministry of health; FF. AA., armed forces. Models were adjusted for detailed area of residence, per capita expenditure quintile, health subsector, age, sex, educational attainment, and survey year. Models were estimated on 94,995 observations with complete information for all included covariates. The main model contrasts are shown.

For waiting >60 min, the territorial pattern was reversed. Compared with Metropolitan Lima, other urban areas did not show a statistically clear difference (aPR 0.96; 95% CI 0.91–1.01), whereas rural areas had a substantially lower prevalence (aPR 0.40; 95% CI 0.37–0.43). The intermediate quintiles Q2, Q3, and Q4 showed modestly higher prevalences than Q1 (aPR between 1.10 and 1.13), whereas Q5 did not show a clear difference (aPR 0.97; 95% CI 0.89–1.05). In the subsector analysis, the Armed Forces/Police had a lower prevalence of waiting >60 min than the Ministry of Health (aPR 0.68; 95% CI 0.56–0.84), whereas EsSalud was at the boundary of no difference (aPR 0.95; 95% CI 0.90–1.00).

For poor access, the pattern again aligned with territorial disadvantage outside Metropolitan Lima. Other urban areas had a higher prevalence than Metropolitan Lima (aPR 1.46; 95% CI 1.15–1.87), and rural areas showed an even stronger association (aPR 2.07; 95% CI 1.59–2.70). No monotonic socioeconomic gradient was observed for this composite outcome, because none of the quintiles showed clear differences relative to Q1. Regarding care subsector, EsSalud had a higher prevalence than the Ministry of Health (aPR 1.65; 95% CI 1.26–2.16), and the Armed Forces/Police showed the largest relative excess (aPR 4.18; 95% CI 2.43–7.17).

### 3.7 Supplementary analysis of non-receipt of medications

Supplementary Table S5 presents the supplementary analysis of non-receipt of medications among 10,583 participants with a medical consultation and available data for this component. Compared with Metropolitan Lima, both other urban areas (aPR 0.67; 95% CI 0.52–0.87) and rural areas (aPR 0.41; 95% CI 0.30–0.57) showed lower prevalence of this outcome. The richest quintile had higher prevalence than the poorest quintile (aPR 1.57; 95% CI 1.05–2.37), whereas EsSalud had lower prevalence than the Ministry of Health (aPR 0.51; 95% CI 0.37–0.69). Because this analysis was conducted using a different clinical denominator, a smaller subsample, and outside the main temporal axis of the study, these findings are presented only as an exploratory supplementary analysis.

## 4 Discussion

### 4.1 Main findings

Among adult users who successfully accessed formal facilities, temporal inequity in access to care in Peru was not a unidirectional phenomenon. The main rural disadvantage occurred before contact with the facility, during the travel stage, whereas the main metropolitan and urban disadvantage appeared once the service had been reached, during the waiting stage. In other words, the access “clock” changed direction depending on the phase evaluated. This observation is important because it challenges approaches that treat access as a single dimension and suggests that different territories bear different temporal costs, even within the same national system.

We also observed that the temporal trajectory was more complex than a simple linear improvement. Geographic accessibility remained relatively stable throughout the period, whereas acceptable waiting time and optimal access improved appreciably through 2022 and then declined in 2023–2024. This pattern is compatible with an incomplete or unstable post-pandemic recovery, although the descriptive design and potential changes in the composition of users across years preclude causal attribution of the trajectory to the post-pandemic period. Likewise, differences by subsector suggest that the temporal care experience varies across organizational channels of care; however, these differences should be interpreted as observed associations rather than causal effects attributable to the subsector.

Because health-care utilization changed during the COVID-19 period, the annual estimates should be interpreted as changes in the observed population of formal-service users rather than as changes in health care need in the total population. Including survey year as a categorical covariate allowed non-linear period variation to be accounted for descriptively, but it cannot separate pandemic-related changes in service organization from changes in who sought and successfully reached formal care.

### 4.2 Comparison with other studies

The finding of a greater travel burden in rural areas is consistent with previous geospatial evidence from Peru. Carrasco-Escobar and colleagues estimated substantially longer travel times for rural areas than for urban areas across facilities of different levels (8). Complementarily, Hernández-Vásquez and colleagues documented that a large proportion of Indigenous communities in Loreto, in the Peruvian Amazon, were more than one hour from the nearest facility (9). Our study is consistent with this literature but adds an analytical advantage: rather than measuring potential geographic accessibility across the territory, it captures the temporal experience reported by users who actually managed to use formal facilities.

Our results also engage with previous studies on service use and access barriers in Peru and other Latin American countries. Houghton and colleagues showed that, even after equity-oriented reforms, socioeconomic gaps persisted in barriers to seeking care, including waiting times, lack of resources, and economic constraints (5). Similarly, Díaz-Ruiz and colleagues documented that the use of medical consultation services in Peru was concentrated in more advantaged groups (11). Our findings do not contradict this evidence; rather, they refine it. They suggest that, once the threshold of entering the system has been crossed, temporal costs may be redistributed and expressed as longer urban waiting, even when initial access remains more difficult for rural and socially vulnerable populations.

The pattern by area and subsector is also compatible with the literature on health-system fragmentation and quality. Carrillo-Larco and colleagues described the Peruvian health system as segmented and fragmented, with heterogeneous provision channels and limited capacity to deliver high-quality care to the entire population (4). Roberti and colleagues, in a comparative study of four Latin American countries, showed that inequalities in coverage and quality remained present within and between subsectors, including in Peru (6). From a governance perspective, Hönger and Montag emphasized how political instability, low investment, and budget underexecution constrain the system's operational capacity (7). Our study aligns with this structural interpretation by showing that the concrete experience of access continues to be mediated by this fragmented institutional architecture.

The greater waiting burden observed in Metropolitan Lima and other urban areas is also consistent with the literature that understands waiting times as an indicator of pressure on supply. McIntyre and Chow argue that prolonged waiting is a form of implicit rationing and a reflection of system stress (3). In Peru, Alarcon-Ruiz and colleagues showed that longer waiting times in ambulatory facilities were associated with lower user satisfaction (10). Our results provide additional empirical support for that interpretation and suggest that, in urban contexts with higher demand and greater care complexity, inequity may be expressed less as physical distance and more as organizational congestion.

However, our data also invite a cautious interpretation of the finding of shorter rural waiting times. A recent qualitative study by Pesantes and colleagues in rural Peru showed that users perceived abandonment, limited institutional responsiveness, and poor continuity of care (16). Therefore, shorter rural waiting time should be interpreted as shorter reported time before receiving care among users who reached the facility, not as evidence of greater clinical timeliness, problem-solving capacity, or quality of care. Shorter waiting times may coexist with lower problem-solving capacity, lower staff availability, or lower demand density. This point is important to avoid simplistic interpretations of the rural–urban gradient.

The supplementary medication analysis should be interpreted within this same cautionary framework. It used a different clinical denominator and captured only whether medications were received, not whether the consultation was clinically appropriate, resolutive, or continuous. Therefore, it does not replace the temporal indicators or provide a direct measure of quality; rather, it illustrates the need to interpret travel and waiting times alongside service-content indicators.

### 4.3 Public health implications

In interpreting these rural findings, the disadvantage should be understood as a structural consequence of territorial organization, infrastructure, and provider-network distribution, rather than as an individual-level deficit among rural users.

First, these findings suggest that equity policies should clearly separate geographic barriers from organizational barriers. In rural areas, where the main disadvantage is concentrated in travel, the most promising interventions are likely to occur outside the consultation room: health transport, strengthening first-level care with greater problem-solving capacity, more agile referral networks, itinerant services, and coordination with local governments to improve roads and connectivity. The regional review by Maceira and colleagues supports the idea that primary care and care networks can function as concrete tools to bring services closer and reduce territorial gaps (17).

Second, the urban disadvantage in prolonged waiting requires equity to be considered also as a problem of supply organization. In metropolitan areas, reducing the time people wait within the system requires redesigning appointment schedules, improving ambulatory triage, redistributing personnel, extending service hours, and strengthening scheduling and follow-up systems. Expanding coverage without active flow management may translate into persistent congestion and, consequently, a form of formal but not timely access.

Third, the observed differences between subsectors indicate that institutional segmentation continues to have tangible consequences for the care experience. In a system where insurance and provision are distributed across networks with different resources and rules, the promise of coverage may not translate into real equality of access. Evidence on insurance-system fragmentation and COVID-19 mortality in Peru also suggests that this institutional architecture may have relevant effects beyond ambulatory access, including hard health outcomes (18). From a health-policy perspective, this reinforces the need for greater institutional interoperability, clearer referral and counter-referral standards, and common performance metrics across subsystems.

These subsector differences should also be interpreted in light of the fact that provider networks are not geographically interchangeable. Differences between MINSA, EsSalud, private providers, and Armed Forces/Police services may reflect not only organizational performance, but also the territorial distribution of eligible facilities, referral rules, and catchment-area constraints. Because ENAHO does not link users to geocoded provider networks, our models adjusted for reported subsector but could not directly model network coverage or distance to the nearest eligible provider.

Fourth, our results have implications for measuring universal health coverage. Traditional indicators of service use or contact with the system do not capture how much time people invest in receiving care. The global report by WHO and the World Bank, together with the literature on high-quality health systems, underscores that coverage expansion must translate into effective access and timely care (1, 2). Incorporating travel and waiting indicators into routine surveillance would make it possible to detect currently invisible inequities and to assess more accurately whether the system is truly moving toward effective coverage.

### 4.4 Limitations

This study has limitations. First, because it was restricted to adults who reported a health problem and successfully attended a formal health care facility, it does not estimate inequities in total population access. Individuals who did not seek care, self-treated, used pharmacies or drugstores, received home care, or used other noninstitutional options may have experienced different, and possibly greater, barriers. Therefore, the findings should be interpreted as temporal costs conditional on formal-service use, not as a measure of access among all people with health care needs. Second, travel and waiting times were self-reported and may be subject to recall error or rounding. Third, 30,427 eligible formal-service users (24.3%) were excluded from the main analytical sample because temporal information was incomplete or recoded as missing. The comparison between included and excluded records showed differences by survey year, area of residence, socioeconomic position, educational attainment, and especially health subsector, with private-sector care strongly overrepresented among excluded records. Therefore, the main estimates should be interpreted as temporal-access estimates among formal-service users with valid travel and waiting time information, rather than as fully representative of all eligible formal-service users; estimates involving the private subsector should be interpreted with particular caution. Fourth, the composite outcome of poor access depended on 60-minute cutoffs, which are useful for comparability and programmatic interpretation but necessarily simplify a continuous access experience and should not be interpreted as universal clinical thresholds. Fifth, the repeated cross-sectional design does not allow causal attribution of observed temporal changes, including changes during and after the COVID-19 period. Finally, although models were adjusted for relevant sociodemographic variables and health subsector, unmeasured factors related to clinical severity, type of consultation, local service availability, appointment modality, seasonality, facility problem-solving capacity, or provider-network configuration may persist.

## 5 Conclusion and recommendations

Among adults who successfully accessed formal health care facilities, temporal inequity operated in opposite directions according to the dimension evaluated and the territory analyzed. Rural disadvantage was concentrated in the phase before contact with the system, with longer travel times than those observed in Metropolitan Lima, whereas the waiting burden was greater in metropolitan and urban settings. This crossed pattern challenges models that conceive of access as a unidirectional rural–urban gradient and indicates that different territories absorb differentiated temporal costs within the same national system. The temporal trajectory reinforced this interpretation: waiting and optimal-access indicators improved through 2022 and then declined in 2023–2024, in a pattern compatible with an incomplete or unstable post-pandemic recovery, although the descriptive design does not allow causal attribution.

From a public health perspective, these findings support the need for policies differentiated according to the dominant barrier in each territory. In rural areas, where the main constraint is geographic, interventions with the greatest potential impact include strengthening the problem-solving capacity of first-level care, expanding itinerant services, financing health transport, and improving road connectivity in coordination with local governments. In metropolitan and urban areas, where the constraint is predominantly organizational, it is necessary to redesign scheduling systems, redistribute the workforce according to demand density, and expand operational capacity to reduce congestion. At the system level, the persistence of differences between subsectors reinforces the need to move toward greater institutional interoperability and common performance metrics. Finally, the routine incorporation of time-based indicators into systems for monitoring effective coverage would allow decision-makers to detect inequities that currently remain invisible.

## Data Availability

Publicly available datasets were analyzed in this study. This data can be found here: https://proyectos.inei.gob.pe/microdatos/.
