# Supplementary material for: Temporal costs of access to formal health care among adult users in Peru: a national analysis of inequalities in travel and waiting times, 2016–2024
Source: Front Health Serv. 2026 Jun 22;6:1864862. doi: 10.3389/frhs.2026.1864862 (PMC13333622; doi:10.3389/frhs.2026.1864862)

# Supplementary Material

**Supplementary Data**

Supplementary Table 1. STROBE checklist for cross-sectional studies.

| **STROBE item** | **Summary recommendation** | **Location in the manuscript** |
| --- | --- | --- |
| 1a | State the study design using a commonly used term in the title or abstract. | Title; Abstract, Methods. |
| 1b | Present an informative and balanced summary of what was done and what was found. | Full abstract. |
| 2 | Explain the scientific background and rationale for the study. | Introduction, paragraphs 1-3. |
| 3 | State specific objectives, including prespecified hypotheses when applicable. | Introduction, final paragraph; Abstract, Objective. |
| 4 | Present the key elements of the study design early. | Methods, Study design and data source. |
| 5 | Describe the setting, location, and relevant dates of data collection. | Methods, Study design and data source; ENAHO 2016-2024 period. |
| 6a | Describe eligibility criteria, participant sources, and selection methods. | Methods, Study population; Supplementary Figure 1. |
| 7 | Clearly define outcomes, exposures, predictors, covariates, and potential confounders. | Methods, Variable construction. |
| 8 | Indicate data sources and measurement methods for each variable of interest. | Methods, Study design and data source; Variable construction. |
| 9 | Describe efforts to address potential sources of bias. | Methods, selection criteria, handling of extreme values and complete cases; Supplementary Table 2; Discussion, Limitations. |
| 10 | Explain how the study size was determined. | Methods, Study population, selection criteria, and definition of the analytical samples. |
| 11 | Explain how quantitative variables and the cut points used were handled. | Methods, Variable construction; 60-minute cut point and handling of values >480 minutes. |
| 12a | Describe the statistical methods, including those used to control for confounding. | Methods, Statistical analysis. |
| 12b | Describe methods for analyzing groups or subgroups, when applicable. | Methods, Statistical analysis; Results by area of residence and subsector. |
| 12c | Explain how missing data were addressed. | Methods, Study population; complete-case comparison in Supplementary Table 2; Results, Participant selection; Discussion, Limitations. |
| 12d | Describe the analytical methods that incorporate the sampling strategy. | Methods, Statistical analysis; use of weights, strata, and clusters. |
| 12e | Describe sensitivity analyses when performed. | Methods, Statistical analysis; no additional sensitivity analyses were performed. |
| 13a | Report the number of individuals at each stage of the study. | Results, Participant selection; Supplementary Figure 1. |
| 13b | State reasons for exclusion or non-inclusion at each relevant stage. | Methods, Study population; Results, Participant selection; Supplementary Figure 1. |
| 13c | Consider use of a flow diagram. | Supplementary Figure 1. |
| 14a | Describe participant characteristics and relevant covariates. | Results, Characteristics of the main sample; Table 1. |
| 14b | Indicate the number of participants with missing data for variables of interest. | Methods, Study population; Supplementary Table 2; Table 1, note on educational attainment; Discussion, Limitations. |
| 15 | Report outcome events or summary measures. | Results; Supplementary Table 3; Supplementary Table 4; Figures 1, 2, and S2. |
| 16a | Present crude and adjusted estimates, their precision, and the variables included in the adjustment. | Results, Adjusted models; Table 2; Supplementary Figure 3. |
| 16b | Report category boundaries when continuous variables were categorized. | Methods, Variable construction; cut points >60 minutes and >480 minutes. |
| 16c | Translate relative estimates into absolute measures when relevant. | Results, weighted prevalences by area; Supplementary Table 3; Figure 2. |
| 17 | Report other analyses performed. | Results, Supplementary analysis of non-receipt of medications; Supplementary Table 5. |
| 18 | Summarize key results in relation to the objectives. | Discussion, Main findings. |
| 19 | Discuss limitations, considering sources of bias or imprecision and their possible direction. | Discussion, Limitations; Supplementary Table 2. |
| 20 | Provide a cautious interpretation considering objectives, limitations, multiplicity, and previous evidence. | Discussion, Comparison with other studies; Implications; Conclusions. |
| 21 | Discuss the generalizability or external validity of the results. | Discussion, Limitations; Conclusions and recommendations. |
| 22 | State the funding source and the role of the funder. | Funding: self-funded study; there was no external funder. |

Note. STROBE: Strengthening the Reporting of Observational Studies in Epidemiology. The checklist is provided for the repeated cross-sectional design of the study; locations are reported by section to avoid relying on page numbers that may change during editorial typesetting.

Supplementary Table 2. Characteristics of eligible formal-service users included in and excluded from the main complete-case analytical sample.

| **Characteristic** | **Category** | **Included n (weighted %)** | **Excluded n (weighted %)** | **Difference, pp** |
| --- | --- | --- | --- | --- |
| Overall | Eligible formal-service users | 95,002 (100.0) | 30,427 (100.0) | 0.0 |
| Survey year | 2016 | 13,999 (13.3) | 3,632 (11.0) | 2.3 |
|  | 2017 | 12,686 (12.9) | 3,372 (11.0) | 1.8 |
|  | 2018 | 13,917 (13.2) | 3,705 (12.2) | 1.0 |
|  | 2019 | 12,926 (13.4) | 3,559 (12.1) | 1.4 |
|  | 2020 | 4,565 (5.0) | 5,041 (14.4) | -9.3 |
|  | 2021 | 6,448 (6.9) | 3,206 (11.2) | -4.3 |
|  | 2022 | 9,240 (10.2) | 2,710 (9.2) | 1.0 |
|  | 2023 | 10,419 (12.2) | 2,692 (9.8) | 2.5 |
|  | 2024 | 10,802 (12.8) | 2,510 (9.1) | 3.7 |
| Area of residence | Metropolitan Lima | 29,593 (48.3) | 14,823 (66.0) | -17.7 |
|  | Other urban | 25,956 (26.2) | 8,272 (21.4) | 4.8 |
|  | Rural | 39,453 (25.5) | 7,332 (12.6) | 12.9 |
| Sex | Female | 59,623 (63.3) | 18,198 (60.1) | 3.3 |
|  | Male | 35,379 (36.7) | 12,229 (39.9) | -3.3 |
| Age group | 18-59 | 62,275 (65.6) | 20,329 (68.7) | -3.1 |
|  | 60+ | 32,727 (34.4) | 10,098 (31.3) | 3.1 |
| Expenditure quintile | Q1 (poorest) | 28,804 (25.1) | 4,767 (12.4) | 12.7 |
|  | Q2 | 21,340 (21.1) | 5,422 (16.6) | 4.5 |
|  | Q3 | 17,379 (19.9) | 5,465 (17.9) | 2.0 |
|  | Q4 | 15,077 (18.2) | 6,440 (22.4) | -4.2 |
|  | Q5 (richest) | 12,402 (15.7) | 8,333 (30.7) | -14.9 |
| Education | No education | 9,218 (8.5) | 1,971 (5.4) | 3.1 |
|  | Primary | 32,712 (31.0) | 7,848 (23.1) | 7.9 |
|  | Secondary | 31,097 (35.8) | 9,290 (32.5) | 3.3 |
|  | Higher | 21,968 (24.6) | 11,317 (39.0) | -14.4 |
| Healthcare subsector | MINSA | 67,590 (67.2) | 2,409 (5.7) | 61.5 |
|  | EsSalud | 24,223 (28.8) | 694 (2.0) | 26.8 |
|  | Private | 2,144 (2.6) | 27,281 (92.2) | -89.6 |
|  | Armed Forces/Police | 1,045 (1.4) | 43 (0.1) | 1.3 |

Note. n values are unweighted counts; percentages incorporate the pooled ENAHO expansion factor. Difference is included minus excluded in percentage points; negative values indicate a higher weighted proportion among excluded records. Eligible formal-service users were adults who reported care in formal health facilities during ENAHO 2016-2024. The main complete-case sample required valid simultaneous travel and waiting time information after recoding temporal values greater than 480 minutes as missing. Educational attainment had 7 missing values among included records and 1 missing value among excluded records; percentages for education were calculated among non-missing records. MINSA: Ministry of Health; FF.AA.: Armed Forces.

Supplementary Table 3. Weighted prevalence of the main outcomes by area of residence.

| **Outcome/area** | **n** | **Weighted prevalence, % (95% CI)** |
| --- | --- | --- |
| Travel >60 min |  |  |
| Metropolitan Lima | 29,593 | 4.0 (3.6–4.4) |
| Other urban | 25,956 | 4.2 (3.9–4.6) |
| Rural | 39,453 | 11.3 (10.7–11.9) |
| Waiting >60 min |  |  |
| Metropolitan Lima | 29,593 | 22.2 (21.5–23.0) |
| Other urban | 25,956 | 21.8 (21.0–22.7) |
| Rural | 39,453 | 9.2 (8.7–9.6) |
| Poor access |  |  |
| Metropolitan Lima | 29,593 | 1.1 (1.0–1.3) |
| Other urban | 25,956 | 1.5 (1.3–1.8) |
| Rural | 39,453 | 1.9 (1.7–2.1) |

Note. n values are unweighted counts; prevalences incorporate the complex sampling design and expansion factors.

Supplementary Table 4. Annual trends in the main access indicators.

| **Year** | **Indicator** | **n** | **Weighted prevalence, % (95% CI)** |
| --- | --- | --- | --- |
| 2016 | Travel ≤60 min | 13,999 | 93.4 (92.8–94.0) |
| 2016 | Waiting ≤60 min | 13,999 | 79.1 (78.0–80.2) |
| 2016 | Optimal access | 13,999 | 74.1 (72.9–75.2) |
| 2017 | Travel ≤60 min | 12,686 | 93.7 (93.1–94.3) |
| 2017 | Waiting ≤60 min | 12,686 | 80.8 (79.7–81.8) |
| 2017 | Optimal access | 12,686 | 75.8 (74.6–77.0) |
| 2018 | Travel ≤60 min | 13,917 | 93.8 (93.2–94.5) |
| 2018 | Waiting ≤60 min | 13,917 | 80.7 (79.5–81.8) |
| 2018 | Optimal access | 13,917 | 75.9 (74.7–77.1) |
| 2019 | Travel ≤60 min | 12,926 | 94.4 (93.8–95.0) |
| 2019 | Waiting ≤60 min | 12,926 | 80.3 (79.2–81.4) |
| 2019 | Optimal access | 12,926 | 76.2 (75.0–77.4) |
| 2020 | Travel ≤60 min | 4,565 | 94.3 (93.3–95.3) |
| 2020 | Waiting ≤60 min | 4,565 | 83.5 (81.7–85.3) |
| 2020 | Optimal access | 4,565 | 79.2 (77.2–81.1) |
| 2021 | Travel ≤60 min | 6,448 | 93.8 (92.9–94.8) |
| 2021 | Waiting ≤60 min | 6,448 | 83.8 (82.2–85.3) |
| 2021 | Optimal access | 6,448 | 79.0 (77.3–80.7) |
| 2022 | Travel ≤60 min | 9,240 | 95.0 (94.4–95.7) |
| 2022 | Waiting ≤60 min | 9,240 | 84.2 (83.0–85.4) |
| 2022 | Optimal access | 9,240 | 80.4 (79.1–81.7) |
| 2023 | Travel ≤60 min | 10,419 | 94.2 (93.4–94.9) |
| 2023 | Waiting ≤60 min | 10,419 | 80.5 (79.3–81.8) |
| 2023 | Optimal access | 10,419 | 76.2 (74.9–77.6) |
| 2024 | Travel ≤60 min | 10,802 | 94.4 (93.8–95.0) |
| 2024 | Waiting ≤60 min | 10,802 | 81.4 (80.2–82.5) |
| 2024 | Optimal access | 10,802 | 77.4 (76.2–78.6) |

Note. n values are unweighted counts; prevalences incorporate the complex sampling design and expansion factors.

Supplementary Table 5. Adjusted prevalence ratios for non-receipt of medications among participants with a medical consultation and available data for this component.

| **Characteristic** | **Non-receipt of medications, aPR (95% CI)** |
| --- | --- |
| Area of residence |  |
| Metropolitan Lima (reference) | 1.00 (ref.) |
| Other urban | 0.67 (0.52–0.87) |
| Rural | 0.41 (0.30–0.57) |
| Per capita expenditure quintile |  |
| Q1 (poorest; reference) | 1.00 (ref.) |
| Q2 | 1.01 (0.70–1.45) |
| Q3 | 1.28 (0.88–1.86) |
| Q4 | 1.29 (0.87–1.90) |
| Q5 (richest) | 1.57 (1.05–2.37) |
| Health subsector |  |
| MINSA (reference) | 1.00 (ref.) |
| EsSalud | 0.51 (0.37–0.69) |
| Private | 0.84 (0.48–1.47) |
| Armed Forces/Police | 0.80 (0.42–1.51) |

Note. aPR: adjusted prevalence ratio; 95% CI: 95% confidence interval; MINSA: Ministry of Health; FF. AA.: Armed Forces. Model adjusted for detailed area of residence, per capita expenditure quintile, health subsector, age, sex, educational attainment, and survey year.

Supplementary Figure 1. Flow diagram of participant selection and analytical branches. The main branch corresponds to time-based outcomes, and the supplementary branch to the exploratory medication analysis.


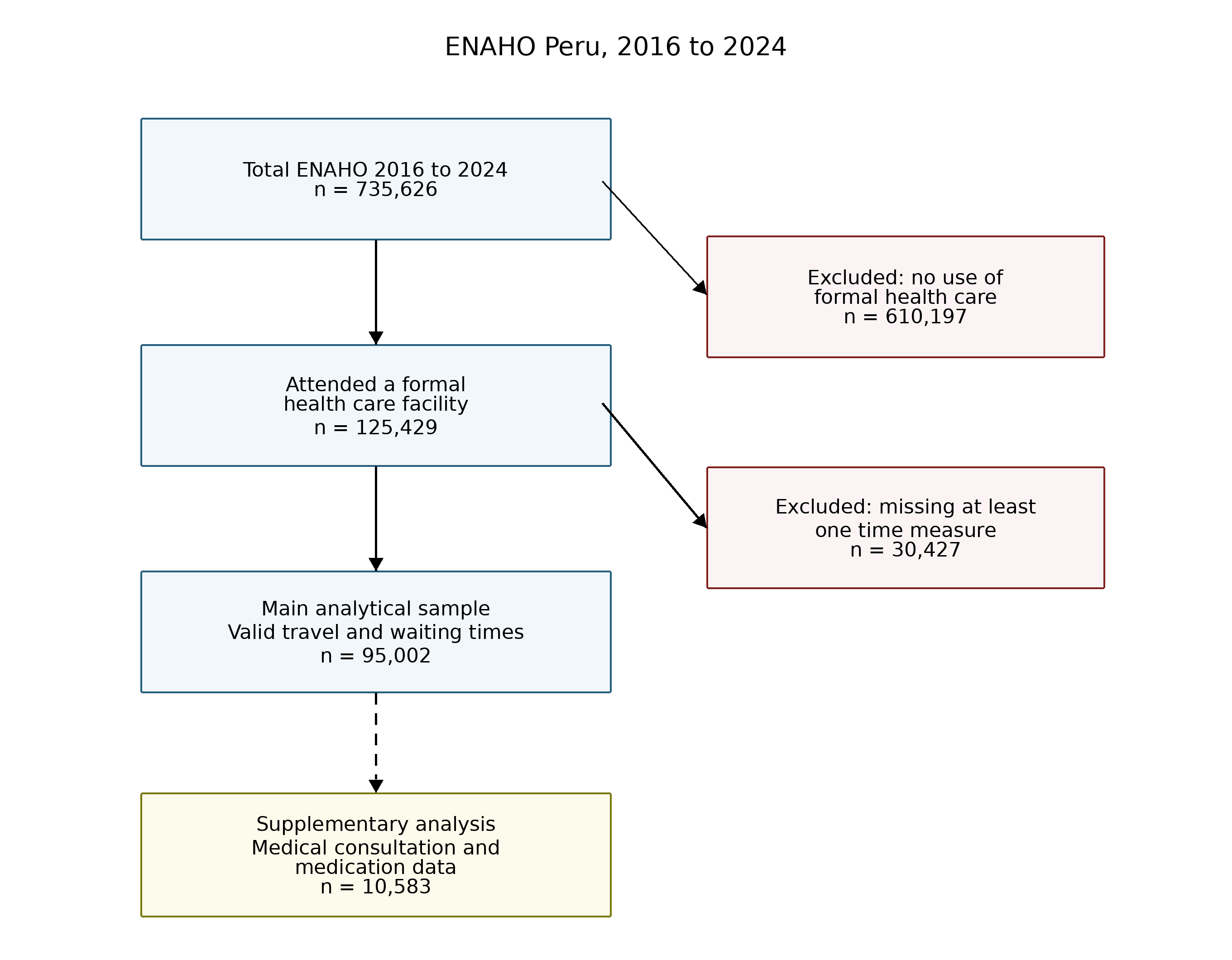


Supplementary Figure 2. Main outcomes by area of residence. Bars show weighted prevalences, and error bars represent 95% CIs.


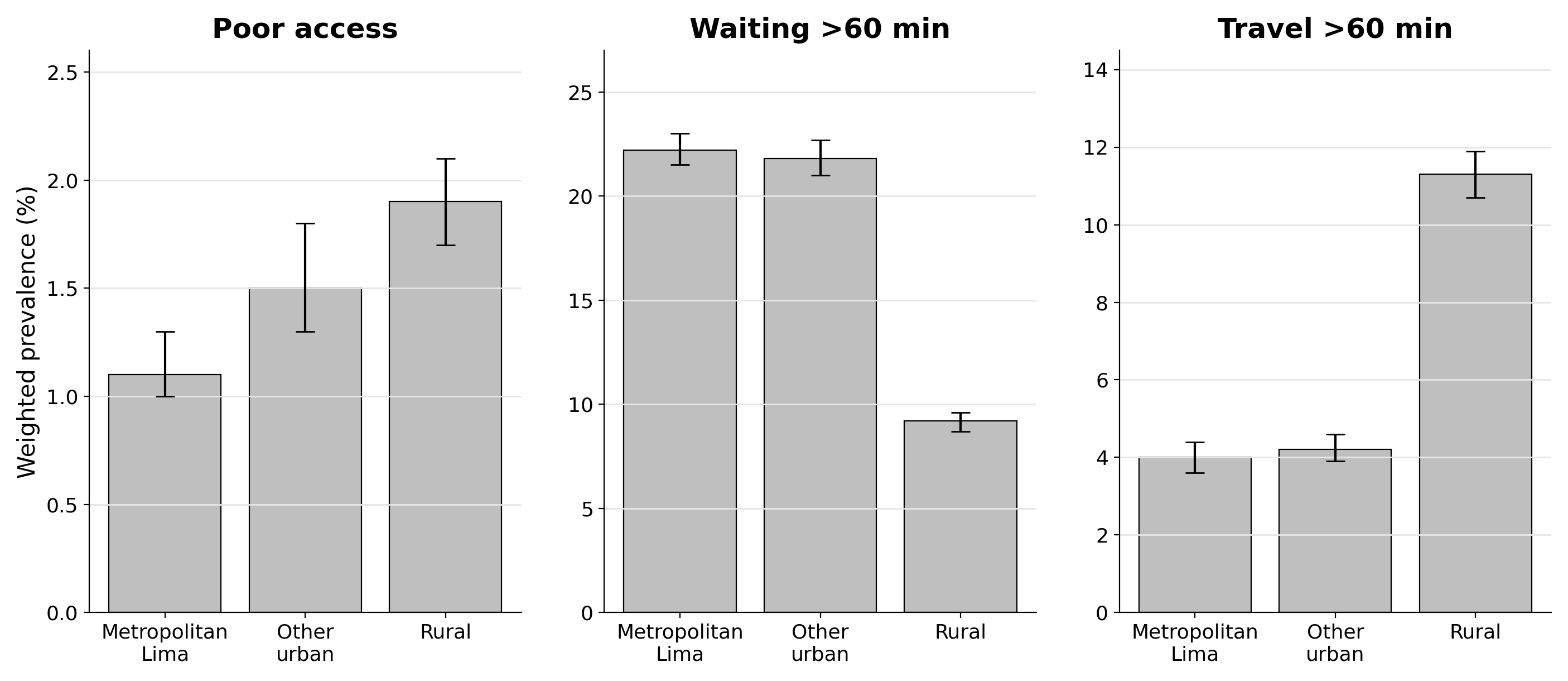


Supplementary Figure 3. Adjusted prevalence ratios for the main outcomes. Points represent aPRs, and horizontal lines represent their 95% CIs; the vertical line indicates the null value.


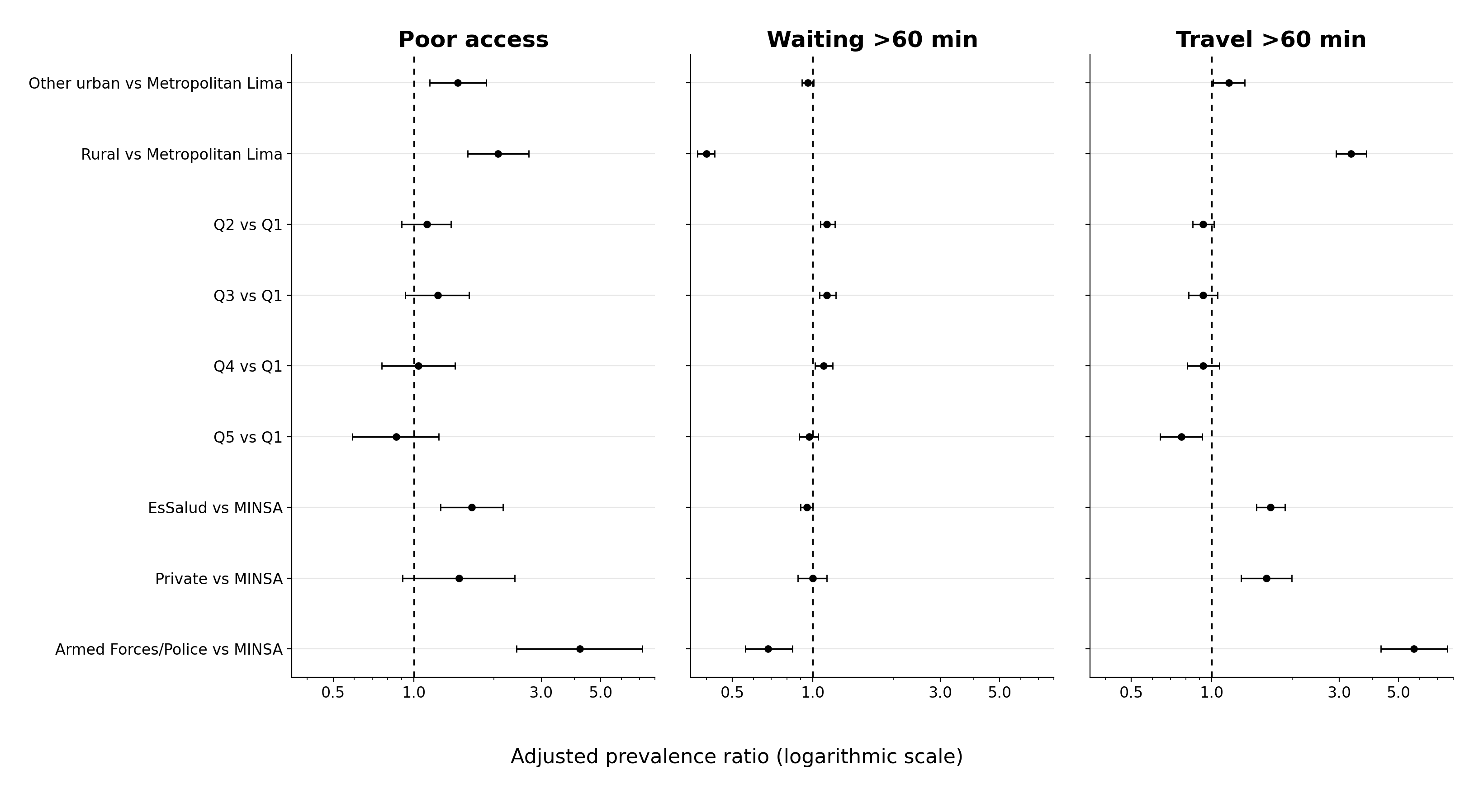

Supplement: Supplementary file 1 [file Table1.docx]
